# A Resident-Centered View of Sexual Expression in Long-Term Care

**DOI:** 10.1093/geroni/igaa057.2033

**Published:** 2020-12-16

**Authors:** Maggie Syme, Tracy Cohn, Allyson Graf

**Affiliations:** 1 Kansas State University, Manhattan, Kansas, United States; 2 Radford University, Radford, Virginia, United States; 3 Northern Kentucky University, Highland Heights, Kentucky, United States

## Abstract

Contrary to societal beliefs, older adults residing in nursing homes continue to have sexual interests. However, the right and need to engage in intimate relationships are often ignored in nursing homes, erring on the side of safety and prohibiting intimacy. This is due to ageist sexual stigma, under-educated providers, complexities of care, and fear of retaliation. Nursing homes are not attending to resident preference/choice when it comes to sexuality and intimacy, which ignores the consumer of services and is wholly inconsistent with person-centered care. However, in order to craft resident-centered/informed policies and practices, we must understand the needs and preferences of the resident/consumer. The following presentation will highlight findings from three separate mixed-methods studies that address the consumer, or how now and future residents understand and evaluate intimacy and sexuality in long-term care. We will address potential challenges, areas for intervention, and a unique consumer-oriented solution— sexual advance directives.

